# Supplementary material for: Sustained-input switches for transcription factors and microRNAs are central building blocks of eukaryotic gene circuits
Source: Genome Biol. 2013 Aug 23;14(8):R85. doi: 10.1186/gb-2013-14-8-r85 (PMC4054853; doi:10.1186/gb-2013-14-8-r85)
Supplement: Additional file 5 — HTML Browsable Motif Output. Zipped folder containing all WaRSwap and FANMOD motif output, viewable in a web browser. [file gb-2013-14-8-r85-S5.ZIP › HTML_browsable_motif_output/WaRSwap_ath_tair10_withSelfLoops/sigs_WSWRSLDraw_TAIR10_SLColors-2500.pvals.heatmaps.html/motif_id_38_010030113_tftype_ath_upstream_-3000_0.html]

```
BG_MODEL = WARSwap
MOTIF_ID = 38_010030113
TF_TYPE = ath
UPSTREAM = -3000_0


PVals
FNR = 0.2	FNR = 0.4	FNR = 0.6	FNR = 0.8
deltaG = 60	0.015	0.001	0.025	0.26
deltaG = 70	0.015	0.002	0.016	0.268
deltaG = 80	0.013	0.002	0.026	0.254

ZScores
FNR = 0.2	FNR = 0.4	FNR = 0.6	FNR = 0.8
deltaG = 60	2.323	3.761	2.393	-0.474
deltaG = 70	2.351	3.672	2.628	-0.472
deltaG = 80	2.333	3.717	2.424	-0.461

StDevs
FNR = 0.2	FNR = 0.4	FNR = 0.6	FNR = 0.8
deltaG = 60	78.63	26.577	7.32	0.974
deltaG = 70	77.661	27.282	6.833	0.996
deltaG = 80	77.656	26.87	7.317	0.985
```
